# Synergistic Activity of Capsaicin and Colistin Against Colistin-Resistant *Acinetobacter baumannii*: In Vitro/Vivo Efficacy and Mode of Action

**DOI:** 10.3389/fphar.2021.744494

**Published:** 2021-09-17

**Authors:** Tingting Guo, Mengying Li, Xiaoli Sun, Yuhang Wang, Liying Yang, Hongmei Jiao, Guocai Li

**Affiliations:** ^1^ Department of Microbiology, School of Medicine, Yangzhou University, Yangzhou, China; ^2^ Jiangsu Key Laboratory of Zoonosis/Jiangsu Co-Innovation Center for Prevention and Control of Important Animal Infectious Diseases and Zoonoses, Yangzhou University, Yangzhou, China; ^3^ Jiangsu Key Laboratory of Integrated Traditional Chinese and Western Medicine for Prevention and Treatment of Senile Diseases, Yangzhou, China; ^4^ Department of Pharmacy, Suzhou Hospital of Integrated Traditional Chinese and Western Medicine, Suzhou, China

**Keywords:** *Acinetobacter* baumannii, capsaicin, colistin, combination, mechanisms

## Abstract

*Acinetobacter baumannii* is an opportunistic pathogen predominantly associated with nosocomial infections. With emerging resistance against polymyxins, synergistic combinations of drugs are being investigated as a new therapeutic approach. Capsaicin is a common constituent of the human diet and is widely used in traditional alternative medicines. The present study evaluated the antibacterial activities of capsaicin in combination with colistin against three unrelated colistin-resistant *Acinetobacter baumannii* strains *in vitro* and *in vivo*, and then further studied their synergistic mechanisms. Using the checkerboard technique and time-kill assays, capsaicin and colistin showed a synergistic effect on colistin-resistant *A. baumannii*. A mouse bacteremia model confirmed the *in vivo* effects of capsaicin and colistin. Mechanistic studies shown that capsaicin can inhibit the biofilm formation of both colistin-resistant and non-resistant *A. baumannii.* In addition, capsaicin decreased the production of intracellular ATP and disrupted the outer membrane of *A. baumannii.* In summary, the synergy between these drugs may enable a lower concentration of colistin to be used to treat *A. baumannii* infection, thereby reducing the dose-dependent side effects. Hence, capsaicin–colistin combination therapy may offer a new treatment option for the control of *A. baumannii* infection.

## Introduction

The emergence of antibiotic resistance poses a serious threat to global health. The overuse and abuse of existing antibiotics have resulted in concerning levels of bacterial resistance and loss of the therapeutic effectiveness of many drugs (Yoshida et al., 2017). *Acinetobacter baumannii* is a non-fermenting Gram-negative opportunistic pathogen that is widely distributed in clinical settings. This bacterium causes a variety of infections in patients with low immunity, including bacteremia and ventilator-associated pneumonia (Wareth et al., 2019). As a result of its propensity to cause outbreaks of infections and develop resistance to most antibiotics, *A. baumannii* has become a serious health threat over the past few decades. Furthermore, clinical multidrug-resistant (MDR) *A. baumannii* isolates are gradually becoming prevalent globally (Magiorakos et al., 2012).

As a result of the lack of effective therapeutic drugs and new antibiotics, some traditional drugs such as polymyxins (colistin, polymyxin B), whose use has been discontinued, have had to be reinstated in the treatment of severe infections caused by MDR Gram-negative bacteria (Bassetti et al., 2013). MDR Gram-negative bacteria are highly sensitive to colistin. Thus, colistin is the last resort for the treatment of severe infections by Gram-negative pathogens, for example infections caused by MDR *Pseudomonas aeruginosa*, carbapenem-resistant Enterobacteriaceae, and MDR *A. baumannii* (Armengol et al., 2019). Unfortunately, with the increased use of colistin, colistin-resistant strains have been detected. To ensure that this last barrier against MDR Gram-negative pathogens remains effective, appropriate action to delay the emergence of resistance and improve the efficacy of antibiotics is urgently needed. Antibiotic adjuvant therapy includes a combination of potent antibiotics and non-antibiotics, which interfere with the mechanism of antibiotic resistance or virulence (Trebosc et al., 2019). This may offer an alternative treatment strategy for infections caused by colistin-resistant *A. baumannii*.

Colistin resistance is conferred by phosphoethanolamine modification of the extracellular membrane, involving phosphoethanolamine transferase PmrC, EptA (Trebosc et al., 2019), and MCR-1 (Rolain et al., 2016). A limitation of colistin is that it can cause severe nephrotoxicity and neurotoxicity. Thus, it is often used in combination with other antibiotics to minimize the dose, and thereby limit its toxicity, while maximizing its antibacterial activity to prevent the emergence of drug resistance (Armengol et al., 2019). In recent years, the synergistic activity of colistin combined with other antibiotics against *A. baumannii* has been explored. Most studies have focused on its combination with carbapenems. The synergistic rate for polymyxin combined with meropenem and doripenem is reported to be approximately 85.0%, which is higher than the synergistic rate with imipenem (66.8%) (Ni et al., 2015). Dinc et al. (2015) studied the effect of colistin combined with doripenem in a mouse model of sepsis induced by carbapenem-resistant *A. baumannii*, and the effects of the combination therapy were superior to those of the single drug treatment group. *In vivo* experiments in a mouse model of XDR *A. baumannii* thigh infection demonstrated that the use of rifampicin significantly increased the efficacy of colistin, substantially reducing the bacterial load during infection in mice (Fan et al., 2016). Furthermore, the synergistic activity between polymyxin and glycopeptides, minocycline, sulbactam, and fosfomycin has also been reported (Claeys et al., 2014; Yang et al., 2016; Leelasupasri et al., 2018; Han et al., 2019).

Compared with synthetic antibiotics, phytochemicals have the characteristics of low toxicity and low cost, which make them a good choice as drugs or adjuvants. Consequently, phytochemicals are increasingly being used as alternative or complementary compounds in combination with conventional antibiotics (Shin et al., 2018). Capsaicin (8-methyl-N-vanillyl-6-nonenamide) and related compounds (capsaicinoids) are secondary metabolites of chili pepper, which play an important role in the immune defense of plants (Marini et al., 2015). Such compounds have important pharmacological and physiological properties such as anti-inflammatory, analgesic, anticancer, and cardioprotective properties, as well as exerting beneficial effects on the gastrointestinal system (Srinivasan, 2016). Additionally, capsaicin has attracted interest in recent years due to its antibacterial and antiviral activities. A recent study showed that capsaicin inhibits the growth of *Vibrio cholerae* and significantly reduces the expression of toxin-associated genes (Erfanimanesh et al., 2019). Another study demonstrated that capsaicin has an inhibitory effect on the growth and biofilm formation of *Porphyromonas gingivalis*, decreasing the secretion of gingivomucosal inflammatory cytokine (Zhou et al., 2014). To date, no reports have been published on the combination of colistin and capsaicin in the treatment of *A. baumannii* infection.

In our study, we detected the synergistic activity of colistin in combination with capsaicin against colistin-resistant *A. baumannii in vitro* and constructed a mouse bacteremia model to assess its *in vivo* activity. Mechanistic study demonstrated that capsaicin can inhibit the biofilm formation, decrease the production of intracellular ATP and disrupt the outer membrane of *A. baumannii*, but has no effect on the inner membrane. Transcription analysis shown that capsaicin can inhibits protein synthesis and efflux activity. Taken together, capsaicin dramatically rescued colistin efficacy on *A. baumannii.*

## Materials and Methods

### Reagents

Colistin and capsaicin were obtained from Selleck (Huston, TX, United States) and Tocris Bioscience (Bristol, United Kingdom), respectively. For *in vitro* studies, capsaicin was dissolved in dimethyl sulfoxide (DMSO; Sigma-Aldrich, St. Louis, MO, United States), and then diluted with culture medium to the desired concentrations. For *in vivo* studies, capsaicin was dissolved in ethanol, and then diluted with normal saline to the required concentration, with a final ethanol concentration of 0.2% (Zhang et al., 2018).

### Bacterial Strains and Susceptibility Test

Two non-clonal MDR clinical *A. baumannii* strains Ab13 and Ab156 isolated at the Affiliated Hospital of Yangzhou University and the type strain ATCC 19606 were used in this study. All of these strains are sensitive to colistin. The distinct antibiotic resistance profiles of Ab13 and Ab156 are shown in Table 1. The minimum inhibitory concentrations (MICs) of colistin and capsaicin against *A. baumannii* were evaluated in triplicate, based on the standard broth microdilution method recommended by the Clinical and Laboratory Standards Institute (CLSI) (Matuschek et al., 2018). The criteria for the interpretation of the susceptibility tests were based on the CLSI guidelines.

**TABLE 1:** Antibiotic resistances of clinical *A. baumannii* strains Ab13 and Ab156. R represent resistance. S represent sensitive.

Strain Antibiotics	Ab13	Ab156
Piperacillin	R	R
Sulbactam	R	R
Tazobactam	R	R
Clavulanic acid	R	R
Ceftazidime	R	R
Cefepime	R	R
Cefotaxime	R	R
Imipenem	R	R
Meropenem	R	R
Gentamicin	R	R
Tobramycin	R	R
Amikacin	R	R
Doxycycline	R	R
Minocycline	**S**	**S**
Tetracycline	R	R
Ciprofloxacin	R	R
Levofloxacin	**S**	R
Gatifloxacin	**S**	**S**
Sulfisoxazole	R	R
Polymyxin B	R	R

### Induction of Colistin-Resistant *A. baumannii*

*A. baumannii* strains Ab13, Ab156, and ATCC 19606 were subcultured in 5 mL of Mueller–Hinton broth (MHB; Oxoid Ltd., Cambridge, United Kingdom) containing colistin (Sigma-Aldrich) at concentrations ranging from 0 to 32 μg/mL for 24 h at 37°C with shaking at 200 rpm. Resistant mutants were selected from MH agar plates containing 10 μg/mL of colistin and were then stored as glycerol stocks at −80°C. The obtained colistin-resistant *A. baumannii* were designated Ab13-R, Ab156-R, and ATCC 19606-R, respectively.

### Checkerboard Assays

Checkboard assays were carried out to test the MIC of the combination of colistin and capsaicin. In brief, increasing concentrations of capsaicin (0–256 μg/mL) in each column and increasing concentrations of colistin (0–64 μg/mL) in each row were set up in 96-well microtiter plates. Each well was inoculated with 100 μL of suspension containing 5 × 10^5^ CFU/mL of the test strain, and then the required concentration of drugs was added to a final volume of 200 μL. The plates were incubated at 37°C for 24 h. Data were obtained from at least three independent experiments.

### Time-Kill Assays

The test strains were subjected to time-kill assays using colistin alone, capsaicin alone or colistin combination with capsaicin. Assays were performed in duplicate. Tubes containing MHB, with or without antibiotic, were inoculated with approximately 5 × 10^6^ CFU/mL log-phase inoculum to a final volume of 5 mL. Then, the tubes were cultured in an ambient atmosphere at 37°C with shaking at 200 rpm. The number of bacteria was determined at time points of 0, 2, 4, 6, 8, and 12 h by enumerating colonies in 10-fold serially-diluted specimens of 100 mL aliquots plated on Mueller–Hinton agar at 37°C. Synergism was defined as a >2 log10 decrease in CFU/mL after 12 h when the combination treatment was used compared with colistin or capsaicin alone.

### Safety Assessment

Hemolytic activity of colistin in the presence of capsaicin was evaluated as follows. 8% sheep blood cells that prepared from fresh sterile defibrinated sheep blood, were treated with various concentrations of colistin (8–32 μg/mL) alone or in combination with capsaicin (8–128 μg/mL) at 37°C. After incubation for 1h, the absorption of supernatant was measured at 576 nm by Infinite M200 Microplate reader (Tecan). PBS (0.01M, pH = 7.4) was used as a negative control, and PBS with Triton X-100 (0.2%) was used as a positive control. Comparing the absorbance of the sample with the positive control is the hemolysis rate.

Cytotoxicity on mammalian cells was performed on A549 cells (adenocarcinomic human alveolar basal epithelial cells)by Cell Counting Kit-8 (CCK-8) assay according to the manufacturer’s instructions (Beyotime, Shanghai, China). A549 cells were seeded in a 96-well plate at about 2 × 10^3^ cells/well and cultured for 20 h. These cells were then treated with capsaicin (8–128 μg/mL) for 24 h, with three replicates for each concentration. After 24 h of culture, 10 μl of CCK-8 solution per well was added. Absorbance was measured at 450 nm to calculate the cytotoxicity of capsaicin.

### Mouse Bacteremia Model

For the *in vivo* experiments, the efficacy of colistin and capsaicin for the treatment of *A. baumannii* infection was determined using the murine bacteremia infection model. Four-week-old BALB/C male mice of approximately 20 g, purchased from the Experimental Animal Center of Yangzhou University (Yangzhou, China), were infected intraperitoneally (i.p.) with a washed overnight culture of *A. baumannii* ATCC19606-R (1 × 10^7^ CFU). After 24 h, the mice were randomly divided into four groups (n = 6 per group) and received daily treatment via i.p. injection: the control group received sterile PBS, the colistin group received 5 mg/kg colistin, the capsaicin group received 3 mg/kg capsaicin, and the combination group received 5 mg/kg colistin and 3 mg/kg capsaicin. Treatment lasted for 3 days. Twenty-4 hours after the last therapeutic dose, mice were euthanized and the tissues (e.g., spleen, kidney, liver, and lungs) were removed, weighed, homogenized, and serially diluted in PBS. The bacterial counts in the target tissues of each group were computed and presented as the mean (±SD) log10 CFU/g tissue or log10 CFU/mL blood.

Mice were handled following the Guidelines for the Care and Use of Laboratory Animals and procedures were approved by the Ethical Committee of Yangzhou University and the Laboratory Animal Management Committee of Yangzhou Province. All animals were acclimated to the controlled environment for 1 week prior to the experiment.

### Biofilm Formation and Quantification

The effects of capsaicin on *A. baumannii* biofilm formation were analyzed using a 96-well tissue culture-treated polystyrene plate. The bacterial cell suspension (1 × 10^6^ CFU/mL) was cultured in wells supplemented with capsaicin (0–128 μg/mL). Negative controls containing MH only were included. The inoculated plates were incubated aerobically at 37°C for 24 h. Then, the planktonic bacteria and remaining capsaicin were discarded and the wells were washed twice with phosphate-buffered saline (PBS) and fixed with paraformaldehyde (4%) for 15 min. After removing the paraformaldehyde and staining with 0.4% (w/v) crystal violet (Sinopharm Chemical Reagent Co. Ltd., Shanghai, China) for 30 min, the cells were finally washed with ddH_2_O until no color was visible in the control well. Aliquots of 95% ethanol were added to each well to dissolve the crystals. The average optical density values at 595 nm (OD_595_) were calculated for the samples. The rate of biofilm inhibition was calculated via the following equation (Wei et al., 2017):

[1-(OD_595_ of cells treated with test agent/OD_595_ of untreated control)]× 100.

The combination of colistin (2 μg/mL) and capsaicin (32 μg/mL) or capsaicin (32 μg/mL) alone on the biofilm formation of colistin-resistant *A. baumannii* were done similar to the above study. Minimum biofilm inhibitory concentration (MBIC) is defined as the lowest concentration of an antimicrobial agent that can inhibit the formation of biofilms, and minimal biofilm eradication concentration (MBEC) is defined as the lowest concentration at which bacteria biofilms failed to regrow (Mi et al., 2016). The MBIC values of capsaicin on *A. baumannii* were done as the following steps: 100 μL of bacterial suspensions (1 × 10^6^ CFU/mL) were transferred to the wells of a flat-bottom 96-well microtiter plate. Capsaicin at different concentrations (0–512 μg/mL) were then added and the plates were incubated at 37°C for 24 h. Then, the consequent steps were done similar to the above study. The MBEC values of capsaicin on *A. baumannii* were the lowest concentration that can prevent visible growth in the biofilm recovery medium after 18 h incubation (Ceri et al., 1999; Lin et al., 2020).

### Biochemical Factors Measurement

Outer membrane permeability:1-N-Phenyl-naphthylamine (NPN) assay was used to assess the outer membrane permeability according to a previously study (Jangra et al., 2018). Due to NPN is a hydrophobic fluorescent probe, the fluorescence of NPN increases when it is transferred from aqueous to hydrophobic environment. The permeability of NPN were tested using 96 well plates (black with clear bottom). After treating the bacteria with different concentrations of capsaicin (0–128 μg/mL) or colistin (0–32 μg/mL) without or with capsaicin (32 μg/mL) for 1 h, 1-N-phenylnaphthylamine (NPN) was added with a final concentration of 10 μM, and the fluorescence intensity were measured with the excitation wavelength of 350 nm and emission wavelength of 420 nm.

Cell membrane integrity:Cell membrane integrity experiments were performed as previously described with some modifications (Jangra et al., 2019). Propidium iodide (PI, Thermo Fisher Scientific), a membrane-impermeable fluorescence dye, was used to detect the cell membrane integrity. After washing bacterial cells three times with normal saline (0.9% NaCl), the cell concentration was normalized to an OD_600_ of 0.1. Then, treat the bacteria cells with different concentrations of capsaicin (0–128 μg/mL) or colistin (0–32 μg/mL) without or with capsaicin (32 μg/mL) for 1 h, the supernatants were collected and PI was added to a final concentration of 10 nM. The fluorescence intensity was measured with the excitation wavelength of 535 nm and emission wavelength of 615 nm.

Total ROS: The levels of reactive oxygen species (ROS) in *A. baumannii* were measured using 2′,7′–dichlorodihydrofluorescein diacetate (DCFH-DA) according to the manufacturer’s instructions (Beyotime, Shanghai, China). With the entering of DCFH-DA into *A. baumannii* cells, DCFH-DA was switched to a membrane-impermeable homologous by cellular esterases or oxidized to a fluorescent form by hydroxyl radical, superoxide, orhydrogen peroxide (H_2_O_2_) (Chen et al., 2020). Resistant strains were loaded with DCFH-DA at a final concentration of 10 μM, and then treated by different concentrations of capsaicin (0–128 μg/mL). Fluorescence intensity was measured with the excitation wavelength of 488 nm and emission wavelength of 525 nm.

Intracellular ATP: Intracellular ATP levels of *A. baumannii* were determined using an Enhanced ATP Assay Kit (Beyotime, Shanghai, China). The *A. baumannii* were cultured overnight and resuspended in PBS, then treated with different concentrations of capsaicin (0–128 μg/mL) for 1 h. After centrifugation, the bacterial precipitates were treated with lysozyme and the supernatants were collected for subsequent determination. The test solutions were added into the 96-well plates and incubated at room temperature for 5 min. The luminescence was detected by multi-function microplate reader (Biotek, United States).

### Scanning Electron Microscopy

ATCC19606-R were treated with colistin (2 μg/mL) or capsaicin (32 μg/mL)or their combination for 4 h and were fixed with 2.5% glutaraldehyde at 4°C for 24 h. Then the bacteria were gradual dehydration with ethanol (30, 50, 70, 80, 90, 95, and 100%). The processed samples were dried using a critical point dryer and coated with a layer of gold-palladium using an ion sprayer, and finally observed with SEM (GeminiSEM 300).

### Transcriptomic Analysis

ATCC19606-R were grown in MHB to the early exponential phase, and treated with colistin (2 μg/mL) alone or in combination of capsaicin (32 μg/mL) for 4 h. After quality inspection of RNA samples, cDNA synthesis, cDNA fragmentation modification and sorting, and library amplification were processed, then, the constructed cDNA library was sequenced using Illumina HiSeqTM. The technical services of this process and subsequent bioinformatics analysis were provided by Nanjing Motif Zhigu Biotechnology Co., Ltd.

### Statistical Analysis

Data were presented as mean ± standard deviation (SD) of three replications. Mean values between groups were compared using the two-tailed Student’s t-test. All statistical analyses were performed using GraphPad Prism version 5.04 (GraphPad Software Inc., San Diego, CA, United States) with a *p* value < 0.05 considered significant.

## Results

### Synergistic Activity of Colistin in Combination With Capsaicin in a Checkerboard Assay

The potency of capsaicin as an antibacterial was initially evaluated against colistin-resistant strains using standard broth dilution assays to determine MICs. The MICs of capsaicin were >512 μg/mL for all of the strains tested, revealing the lack of direct antimicrobial activity of capsaicin alone against the ATCC 19606-R, Ab13-R, and Ab156-R colistin-resistant strains.

To explore the interaction of capsaicin and colistin, a checkerboard assay was performed to determine the FIC indices for the laboratory-generated colistin-resistant *A. baumannii*. Checkerboard assays revealed the obvious dose-dependent synergistic activity of capsaicin with colistin on the ATCC 19606-R (FICI = 0.03), Ab13-R (FICI = 0.06), and Ab156-R (FICI = 0.04) colistin-resistant strains (Table 2). Capsaicin could decrease the colistin MICs to values lower than the susceptibility breakpoint (2 μg/mL) in most cases (Table 2). These results suggested that colistin-resistant *A. baumannii* strains have recovered or enhanced sensitivity to colistin when combined with different concentrations of capsaicin.

**TABLE 2:** Colistin MICs of wild-type and laboratory-generated colistin-resistant strains when treated with different concentrations of capsaicin.

Strain	Colistin MIC (μg/mL) at different Capsaicin concentrations (μg/mL)										
	Only colistin	Capsaicin (256)		Capsaicin (128)		Capsaicin (64)		Capsaicin (32)		Capsaicin (16)	
		MIC	Fold change	MIC	Fold change	MIC	Fold change	MIC	Fold change	MIC	Fold change
ATCC19606	0.5	–	–	–	–	–	–	–	–	–	–
ATCC19606-R	1,024	0.5	2048	0.5	2048	0.5	2048	0.5	2048	8	128
Ab13	1	–	–	–	–	–	–	–	–	–	–
Ab13-R	128	1	128	4	32	4	32	4	32	16	8
Ab156	2	–	–	–	–	–	–	–	–	–	–
Ab156-R	64	0.5	128	0.5	128	0.5	128	0.5	128	4	16

### Synergistic Activity of Capsaicin and Colistin in Time-Kill Assays

**FIGURE 1:**
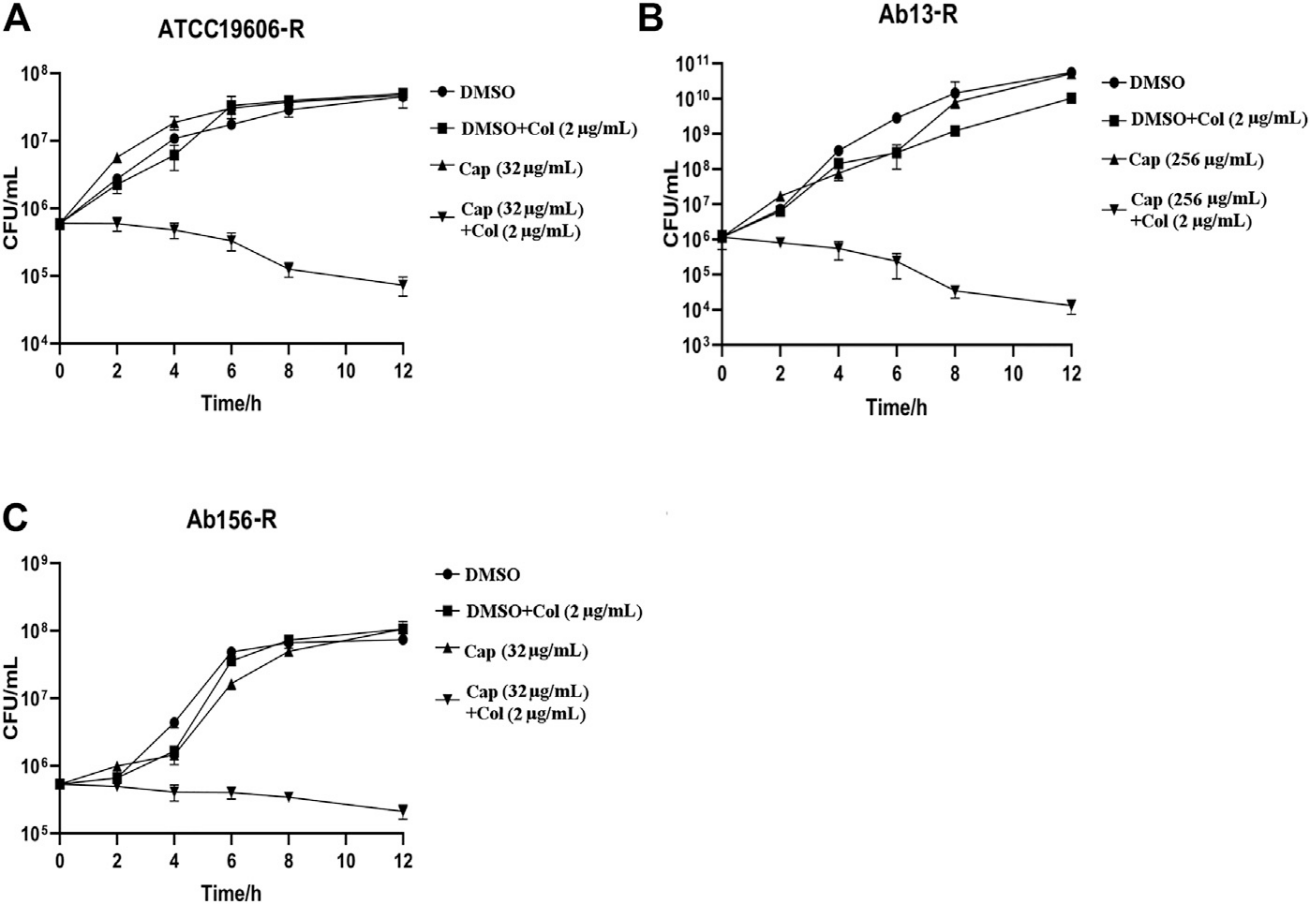
Time-kill curves of *A. baumannii* isolates using the drugs alone or in combination. The capsaicin concentrations of ATCC19606-R, Ab13-R and Ab156-R were 32 μg/mL, 256 μg/mL, 32 μg/mL, respectively; at which the MIC values of colistin are below the susceptibility breakpoint (2 μg/mL). **(A)**: Time-kill curves of ATCC19606-R using capsaicin (32 μg/mL) or colistin (2 μg/mL) without or with capsaicin (32 μg/mL). **(B)**: Time-kill curves of Ab13-R using capsaicin (256 μg/mL) or colistin (2 μg/mL) without or with capsaicin (256 μg/mL). **(C)**: Time-kill curves of Ab156-R using capsaicin (32 μg/mL) or colistin (2 μg/mL) without or with capsaicin (32 μg/mL). Abbreviations: Cap, capsaicin; Col, colistin; DMSO, dimethyl sulfoxide.

To further confirm the synergism of capsaicin with colistin, time-kill assays were carried out with the three colistin-resistant *A. baumannii* strains. The colistin concentration used corresponded to the clinical breakpoint for susceptibility (2 μg/mL), and the capsaicin concentration used for Ab13-R was 256 μg/mL, while that used for Ab156-R and ATCC19606-R was 32 μg/mL, which could inhibit the growth of most bacteria when combined with colistin (Table 2). As shown in Figure 1 A, B, and C, three colistin-resistant strains grew normally within 12 h of using the drug alone. Neither capsaicin nor colistin monotreatment killed colistin-resistant A. baumannii. However, the combination led to a reduction of bacterial load approximate >2 log10 (Figure 1). The above results showed that capsaicin indeed drastically enhanced the effect of colistin against colistin-resistant *A. baumannii*.

### Safety Evaluation of Capsaicin

**FIGURE 2:**
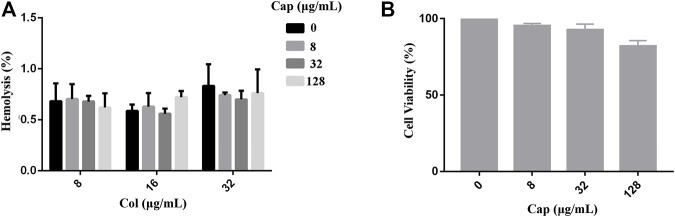
Safety assessment of capsaicin. **(A)**: Hemolytic activity of capsaicin (8–128 μg/mL) in the absence or presence of colistin (8–32 μg/mL) to the RBCs. **(B)**: *In vitro* mammalian toxicity in A549 cell lines. The data shown here are mean ± SD of three replicates.

To evaluate the safety of capsaicin, we performed toxicity tests in red blood cells (RBCs) and mammalian cells. As shown in Figure 2 A, we found that capsaicin (≤128 μg/mL) had negligible effects on the hemolysis of colistin to red blood cells (RBCs). The results of CCK-8 assay shown that capsaicin (≤32 μg/mL) had no cytotoxicity to A549 cells (Figure 2B). 128 μg/mL capsaicin has a lower cell viability of 82% (Figure 2B), which is different with the results of hemolytic activity. Thus, the use of capsaicin is relatively safe under the concentrations of 128 μg/mL.

### Therapeutic Efficacy of Colistin in Combination With Capsaicin in the Murine Model of Bacteremia

**FIGURE 3:**
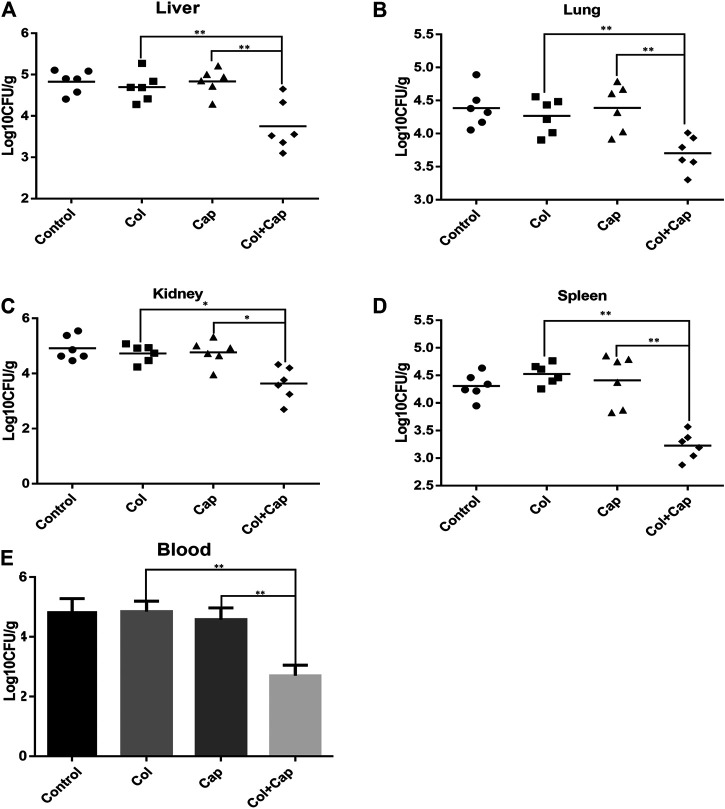
*A. baumannii* burden in the target tissues [**(A)**: liver, **(B)**: lung, **(C)**: kidney, **(D)**: spleen] were quantified as CFU per gram of target tissue in the murine bacteremia model following different regimens. E: Number of *A. baumannii* contained within the blood. The control group received sterile PBS, the colistin group received 5 mg/kg colistin, the capsaicin group received 3 mg/kg capsaicin, and the combination group received 5 mg/kg colistin and 3 mg/kg capsaicin. **p < 0.01 was determined using the Student’s t-test. At least six mice per group were analyzed.

Lethal bacteremia is a common outcome of *A. baumannii* infection so to determine the potential use of combination therapy in clinical situations, we investigated the *in vivo* efficacy of capsaicin combined with colistin in a bacteremia model infected with colistin-resistant *A. baumannii*. In preliminary experiments, we found that, by 24 h, all organs in the mice were heavily infected with *A. baumannii*, suggesting that the infection was systemic. Furthermore, the combined treatment group showed reduced *A. baumannii* densities in the target tissues compared with the untreated or COL/CAP alone groups (Figure 3).

### Effect of Capsaicin on Biofilm Formation

**FIGURE 4:**
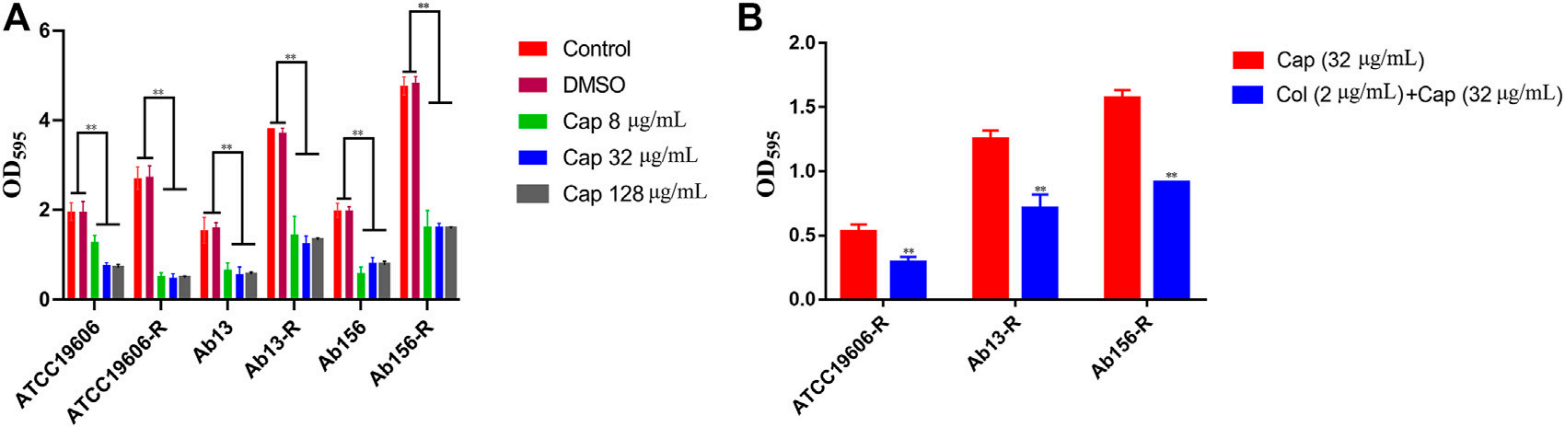
**(A)**: Effect of capsaicin (0–128 μg/mL) on biofilm formation of *A. baumannii.***(B)**: The combination of colistin (2 μg/mL) and capsaicin (32 μg/mL) on the biofilm formation of *A. baumannii.* *p < 0.05, **p < 0.01, data are expressed as the mean ± SD of three replicates.

All of the strains used in this study have the ability to form biofilm. Considering the synergistic effects of capsaicin on colistin observed above, we hypothesized that capsaicin might inhibit biofilm formation of *A. baumannii* and this was tested using 8, 32 and 128 μg/mL of capsaicin. As shown in Figure 4, capsaicin could suppress the biofilm formation of all test strains. Interestingly, the amount of biofilms formed by colistin-resistant strain ATCC19606-R, Ab13-R, and Ab156-R were significantly increased compared with corresponding sensitive strains, suggesting that colistin resistance was positively correlated with biofilm formation in this strain. This provides insight into the potential coevolution of antimicrobial resistance and biofilm formation in Gram-negative pathogens. The MBIC values of capsaicin on ATCC19606, ATCC19606-R, Ab13 and Ab156 were 256 μg/mL. The MBIC values of capsaicin on Ab13-R and Ab156-R were 512 μg/mL. The MBEC values of capsaicin on ATCC19606, ATCC19606-R, Ab13 and Ab156 were 512 μg/mL, and the MBEC values of capsaicin on Ab13-R and Ab156-R were higher than 512 μg/mL. The MBIC and MBEC values of ATCC19606, ATCC19606-R, Ab13 and Ab156 were lower than Ab13-R and Ab156-R. This could be due to the differences in the ability to form biofilms of the bacterial strains tested.

### Capsaicin Destroys the Outer Membrane

**FIGURE 5:**
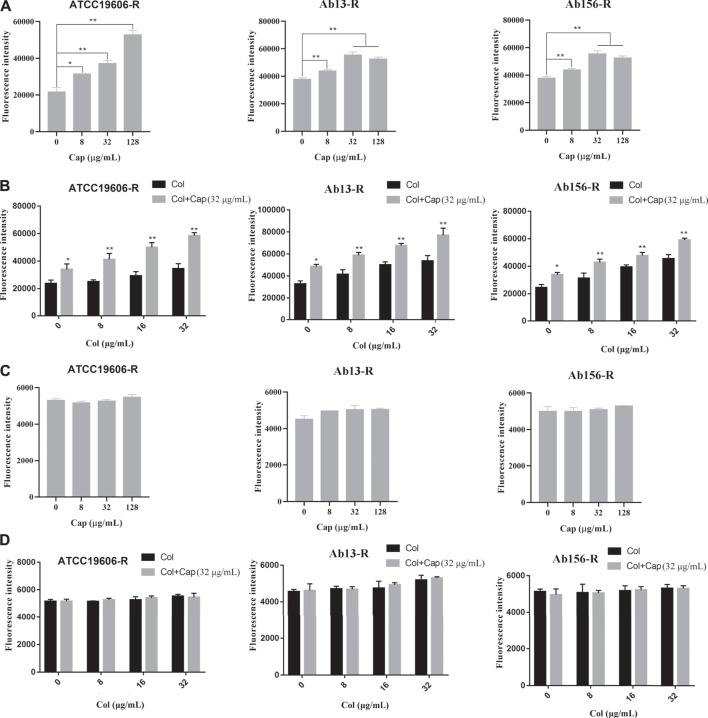
Effect of capsaicin on the permeability of bacterial membrane. **(A, B)**: Capsaicin disrupts the outer membrane of *A. baumannii* ATCC19606-R, AB13-R and AB156-R by measuring fluorescence intensity of 1-N-phenylnaphthylamine (NPN).**(C, D)**: No effect on membrane permeability for propidium iodide (PI) in *A. baumannii* ATCC19606-R, AB13-R and AB156-R after treatment with capsaicin. Bacteria cells were treated with different concentrations of capsaicin (0–128 μg/mL) or colistin (0–32 μg/mL) without or with capsaicin (32 μg/mL).

We further measured the changes of cell membrane permeability after capsaicin treatment. NPN (1-N-phenylnaphthylamine) was used to study the outer membrane permeability of three resistant strains after capsaicin treatment. Due to the experiments calibrated in the same way, the fluorescence intensities showed in Figure 5 A and B were comparable. Figures 5A,B showed that the addition of capsaicin significantly increased outer membrane permeability in a concentration dependent manner. PI (propidium iodide) is often used to evaluate the integrity of inner membrane. As shown in Figures 5C,D, there was no significant changes in fluorescence intensity among the three resistant strains exposed to capsaicin alone or combination with colistin. Similarly, the fluorescence intensities showed in Figures 5C,D were also comparable. These results indicated that capsaicin destroyed the integrity of outer membrane, thus increasing the membrane-damaging ability of colistin, but had no effect on the integrity of inner membrane.

### Capsaicin Reduces Intracellular ATP Content

**FIGURE 6:**
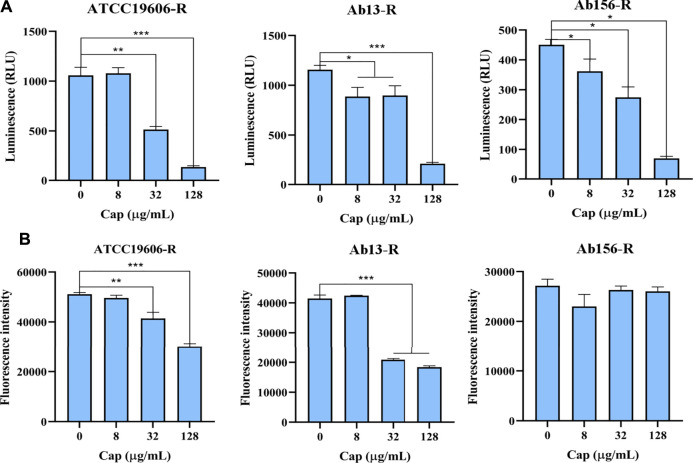
**(A)**: Intracellular ATP in bacterial cells treated with increasing concentrations of capsaicin (0–128 μg/mL). **(B)**: The changes of total ROS of *A. baumannii* ATCC19606-R, AB13-R and AB156-R detected by DCFH-DA fluorescent probe after treatment with capsaicin (0–128 μg/mL).

The effect of capsaicin on intracellular ATP level was determined by luciferase bioluminescence method. As shown in Figure 6A, capsaicin significantly decreased intracellular ATP level in a dose-dependent manner. We speculated that the decrease of intracellular ATP level is related to the increase bactericidal effect of colistin.

### The Antioxidant Effect of Capsaicin

The membrane damage is related to the production of ROS and affects the content of ATP, and the bacteria will produce oxidative damage under antibiotic stress. Therefore, we speculate that capsaicin may increase oxidative damages. As shown in Figure 6B, capsaicin decreased the ROS levels of ATCC19606-R and Ab13-R, while in Ab156-R strain, capsaicin had no effect on ROS levels. It shows that capsaicin mainly plays its antioxidant activity here, though there are differences in different strains.

### The Morphological Changes of *A. baumannii* Under Capsaicin Treatment

**FIGURE 7:**
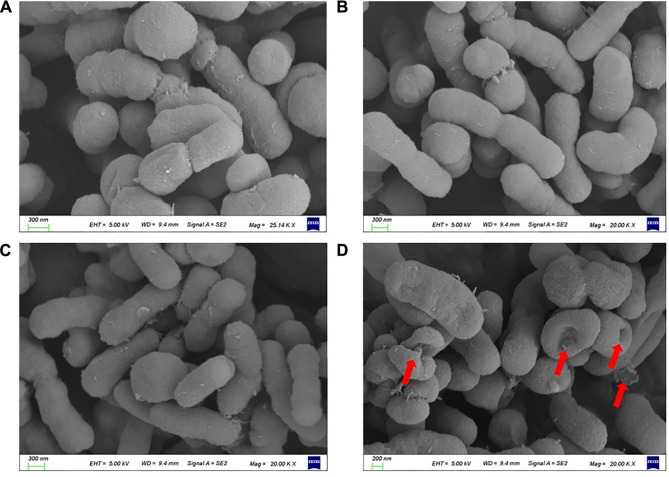
Morphological changes of ATCC19606-R after different treatments. **(A)**: Untreated. **(B)**: Colistin (2 μg/mL) alone treatment. **(C)**: Capsaicin (32 μg/mL) alone treatment. **(D)**: Colistin (2 μg/mL) combined with capsaicin (32 μg/mL).

The resistance of colistin is mainly mediated by the decrease of affinity between colistin and LPS. Therefore, we speculate that capsaicin can restore the ability of colistin to destroy the outer membrane. In order to verify this hypothesis, scanning electron microscopy (SEM) was used to observe the morphological changes of ATCC19606-R after capsaicin treatment. As shown in Figure 7, the surface of cells treated colistin combination with capsaicin showed depression, shrinkage and even collapse and lysis compared with monotreatment, indicating that the bactericidal effect of capsaicin may be related to the rapid destruction of cell surface structure.

### Capsaicin Inhibits Protein Synthesis and Efflux Activity

**FIGURE 8:**
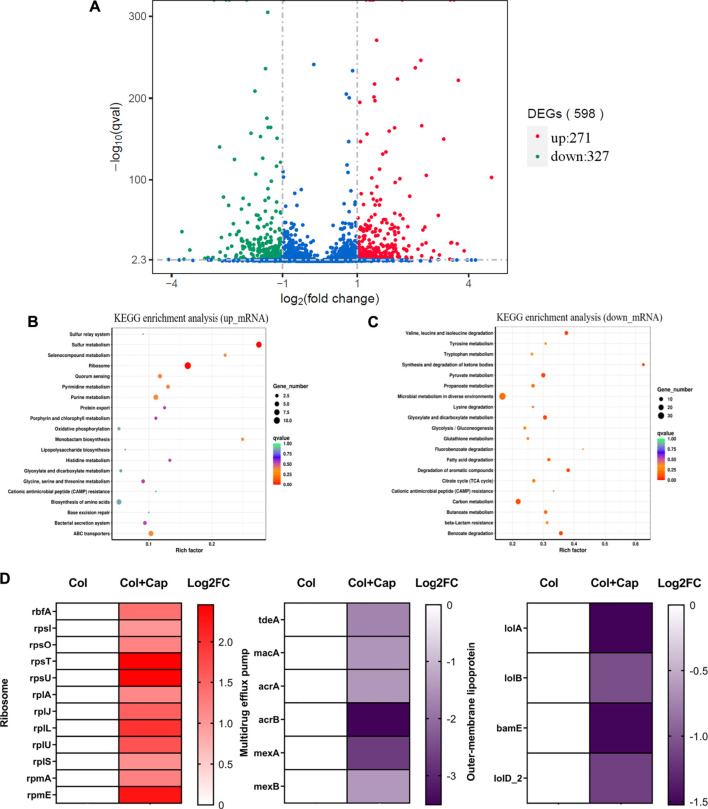
Transcriptome analysis of ATCC19606-R after exposure to colistin (2 μg/mL) alone or the combination of colistin (2 μg/mL) plus capsaicin (32 μg/mL). **(A)**: Differential gene volcano map. **(B)**: KEGG enrichment analysis of up-regulation genes. **(C)**: KEGG enrichment analysis of down-regulation genes; the color of the dot represents the size of the Q value, and the size of the dot represents the number of differential genes. **(D)**: Differentially expressed genes of ribosomes, multidrug efflux pump and outer membrane lipoprotein. Col: colistin alone; Col + Cap: the combination of colistin and capsaicin.

To gain a deeper understanding of the synergy mechanisms of capsaicin, we performed transcription analysis of ATCC19606-R after exposure to colistin or colistin-capsaicin combination for 4 h. The results showed that compared with colistin alone group, the expression of 271 genes in ATCC19606-R were up-regulated and 327 genes were down regulated after 4 h treatment with capsaicin and colistin (Figure 8A). KEGG enrichment analysis showed that the up-regulated genes were significantly enriched in ribosome synthesis (Figure 8B), while the down regulated genes were involved in microbial metabolism (Figure 8C). In addition, the 30S and 50S subunit synthesis-related genes were up-regulated under the combined treatment, which may be due to the inhibition of protein synthesis by capsaicin that was compensated by the up-regulation of ribosomal synthesis genes. The expression of multidrug efflux related genes were significantly down regulated, which indicated that the efflux pumps function of bacteria were inhibited under combined treatment. In addition, the expression of outer membrane protein related-genes also showed decreased, and these proteins were related to the ability of adhesion, invasion, colonization and proliferation (Figure 8D).

## Discussion

*Acinetobacter* spp. are glucose-non-fermentative, aerobic, Gram-negative coccobacilli. The ability of *A. baumannii* to form biofilms on the surface of inanimate objects enables *A. baumannii* to survive in hospital environments, and frequently cause pneumonia and septicemia in immunocompromised patients. *A. baumannii* possesses chromosomal genetic elements that confer resistance against many classes of antibiotics, as well as allowing for survival on inanimate surfaces for prolonged periods of time and strong tolerance to desiccation (Pourhajibagher et al., 2016). Carbapenem-associated MDR *A. baumannii* have been increasingly reported, and the treatment options for infections caused by this pathogen are severely limited. Colistin is currently one of the few antibiotics that remains active against this pathogen, and is considered the last resort treatment for severe Gram-negative infections (Kaye et al., 2016). However, treatment failures with colistin monotherapy and the emergence of drug resistance have promoted the search for combination therapy that synergistically kills colistin susceptible and resistant organisms (Thomas et al., 2019). A promising alternative strategy is provided by non-antibiotic drugs that can overcome resistance mechanisms when combined with failing antibiotics (Brown, 2015).

Many alternative therapies have been studied and amongst these, medicinal plants with various antimicrobial properties, have received significant attention. Furthermore, there are few reports of antimicrobial resistance to these phytochemicals, possibly because of the diversity of their mechanisms of action. In fact, quinones, tannins, terpenoids, alkaloids, flavonoids, and polyphenols are all valuable secondary metabolites that are used by plants in the defense against predation by herbivorous insects and microorganisms (Subramani et al., 2017). The antimicrobial activity and mechanisms of action of these plant-derived compounds have been reported, and they exert their effects through virulence factors that are critical for pathogenicity (Upadhyay et al., 2014).

Capsaicin, found in capsicum fruits, is the predominant, naturally-occurring alkamide (Basith et al., 2016). Capsaicin has a wide range of biological activities, which include anti-oxidant, analgesic, antimicrobial, and anticancer activities, as well as acting against high cholesterol levels and obesity. In this study, we demonstrated that capsaicin can function as an antibiotic adjuvant *in vitro* and *in vivo*, potentiating colistin activity against colistin-resistant *A. baumannii*. Because of the high rates of antibiotic resistance and the resulting treatment failures*, A. baumannii* bacteremia is becoming increasingly challenging to treat and is associated with a high mortality rate (Xiong et al., 2019). In the murine model of bacteremia, capsaicin–colistin combination therapy significantly reduced the bacterial load in the target tissues compared with the untreated control and the colistin/capsaicin monotherapy groups.

*A. baumannii* clinical isolates possess a strong ability to form biofilms. Biofilms are formed when microbial cells become surrounded by self-produced exopolysaccharide matrices on the surfaces of biotic or abiotic surfaces (Eze et al., 2018). Within a biofilm community, *A. baumannii* is more tolerant to extracellular stressors (Harding et al., 2018); hence, higher doses of antibiotics are needed to treat an infection involving biofilm than for planktonic cells, with the resulting risk of antibiotic resistance leading to increased treatment failures. In this study, we found that capsaicin has a significant inhibitory effect on the biofilms of both sensitive and resistant strains, which was consistent with a previous report by Zhou et al. (2014). Therefore, we supposed that the synergistic effect of capsaicin on colistin is partly due to its inhibition of biofilm, which weakens the aggregation of bacterial cells and alleviates the barrier of drug diffusion, enhancing the antibacterial effect of colistin.

The plant antimicrobial agents play a synergistic role in a variety of ways, which are crucial to the normal cell function or virulence. Although the exact mechanisms of antibacterial action of plant antimicrobial agents are still unclear, some potential mechanisms have been reported, such as destruction of bacterial cell membrane, loss of membrane potential, damage of ATP production and inhibition of the expression of virulence genes. When combined with antibiotics, some phytochemicals not only have direct antibacterial activity, but also show synergistic effect *in vitro*, thus changing the antibiotic resistance (Baskaran et al., 2016; Barbieri et al., 2017). Chelation of metal ions, inhibition of membrane-bound ATPase and changes of membrane permeability caused by plant compounds affect the normal physiological function of bacteria and lead to cell death (Subramani et al., 2017). Plant derived antimicrobial agents, such as resveratrol, coumarin, carvacrol, thymol, eugenol and catechins, play an antibacterial role by inhibiting the formation of biofilm and the ability to adhere to the host and destroying the cell membrane (Chen et al., 2016; Li et al., 2016). Essential oil is a secondary metabolite produced by aromatic plants and has significant antibacterial activity. It can interact with multiple targets, resulting in destruction of cell membrane, inhibition of protein synthesis and efflux pump activity (Mittal et al., 2019).

In our study, we found that capsaicin can significantly enhance the sensitivity of *A. baumannii* to colistin. On the one hand, capsaicin inhibits the biofilm formation of *A. baumannii* and destroys the outer membrane permeability, which may enable hydrophobic capsaicin molecules to pass through the lipopolysaccharide in the outer layer of bacteria and reach the target, thus playing a synergistic role. In addition, capsaicin promotes the expression of ribosomal synthesis genes, which is supposed to be the result of its inhibition of protein synthesis. At the same time, capsaicin can also inhibit the expression of drug efflux pump genes and the outer-membrane lipoprotein genes, which meant that the efflux, proliferation and adhesion ability of bacteria were weakened.

## Conclusion

In conclusion, we report that the combination of capsaicin with colistin may be a viable alternative treatment option to combat colistin-resistant *A. baumannii* strains. This treatment reduces the dosage of colistin, thereby reducing the selective pressure on the target strains. One limitation of our study was that the clinical isolates collected did not include isolates that were naturally highly resistant to colistin. The discovery of capsaicin as a new colistin adjuvant highlights the huge potential of natural drug to treat bacterial infections. This may confirm the potential efficacy of capsaicin to treat clinical colistin-resistant *A. baumannii*.

## Data Availability

The data presented in the study are deposited in the NCBI Sequence Read Archive (SRA), accession number SRR15633557 and SRR15633558.
